# Limitations of Species Delimitation Based on Phylogenetic Analyses: A Case Study in the *Hypogymnia hypotrypa* Group (Parmeliaceae, Ascomycota)

**DOI:** 10.1371/journal.pone.0163664

**Published:** 2016-11-09

**Authors:** Xinli Wei, Bruce McCune, H. Thorsten Lumbsch, Hui Li, Steven Leavitt, Yoshikazu Yamamoto, Svetlana Tchabanenko, Jiangchun Wei

**Affiliations:** 1 State Key Laboratory of Mycology, Institute of Microbiology, Chinese Academy of Sciences, Beijing, China; 2 Department of Botany and Plant Pathology, Oregon State University, Corvallis, Oregon, United States of America; 3 Science & Education, The Field Museum, Chicago, Illinois, United States of America; 4 University of Chinese Academy of Sciences, Beijing, China; 5 Department of Biology, Brigham Young University, Provo, Utah, United States of America; 6 Department of Bioproduction Science, Faculty of Bioresource Sciences, Akita Prefectural University, Akita, Japan; 7 Sakhalin Botanical Garden, Yuzhno-Sakhalinsk, Russia; University of Szeged, HUNGARY

## Abstract

Delimiting species boundaries among closely related lineages often requires a range of independent data sets and analytical approaches. Similar to other organismal groups, robust species circumscriptions in fungi are increasingly investigated within an empirical framework. Here we attempt to delimit species boundaries in a closely related clade of lichen-forming fungi endemic to Asia, the *Hypogymnia hypotrypa* group (Parmeliaceae). In the current classification, the *Hypogymnia hypotrypa* group includes two species: *H*. *hypotrypa* and *H*. *flavida*, which are separated based on distinctive reproductive modes, the former producing soredia but absent in the latter. We reexamined the relationship between these two species using phenotypic characters and molecular sequence data (ITS, *GPD*, and *MCM7* sequences) to address species boundaries in this group. In addition to morphological investigations, we used Bayesian clustering to identify potential genetic groups in the *H*. *hypotrypa*/*H*. *flavida* clade. We also used a variety of empirical, sequence-based species delimitation approaches, including: the “Automatic Barcode Gap Discovery” (ABGD), the Poisson tree process model (PTP), the General Mixed Yule Coalescent (GMYC), and the multispecies coalescent approach BPP. Different species delimitation scenarios were compared using Bayes factors delimitation analysis, in addition to comparisons of pairwise genetic distances, pairwise fixation indices (F_ST_). The majority of the species delimitation analyses implemented in this study failed to support *H*. *hypotrypa* and *H*. *flavida* as distinct lineages, as did the Bayesian clustering analysis. However, strong support for the evolutionary independence of *H*. *hypotrypa* and *H*. *flavida* was inferred using BPP and further supported by Bayes factor delimitation. In spite of rigorous morphological comparisons and a wide range of sequence-based approaches to delimit species, species boundaries in the *H*. *hypotrypa* group remain uncertain. This study reveals the potential limitations of relying on distinct reproductive strategies as diagnostic taxonomic characters for *Hypogymnia* and also the challenges of using popular sequence-based species delimitation methods in groups with recent diversification histories.

## Introduction

Molecular sequence data have had a pronounced effect on our understanding of species boundaries, especially in organisms with relatively simple morphologies and considerable variability of phenotypic characters, such as lichen-forming fungi. Similar to most major biological groups, identifying the appropriate character sets is one of the greatest challenges with empirical species delimitation in lichen-forming fungi [1–6]. However, fungi generally have few taxonomically informative traits, in comparison to other major clades of life, and intraspecific variation makes accurate taxonomic circumscriptions more difficult. Hence, molecular data now play a prominent role in circumscribing fungal species. Cryptic species are often identified using molecular data, and in some cases cryptic species are corroborated by formerly overlooked phenotypic characters [7–11]. In other cases, some species-level lineages were shown to consist of chemically or morphologically polymorphic individuals that were previously regarded as separate taxa [12–14].

Differences in reproductive strategies have traditionally played an important role in circumscribing lichen-forming fungal species, with populations forming asexual diaspores (such as powdery soralia or corticated isidia) being separated at the species level from populations lacking those and exhibiting ascomata [15,16]. However, this classification has been challenged [17,18] and several molecular studies have shown that the taxonomic importance of reproductive mode has probably been over-emphasized in a number of fungal groups [3,19–23]. This is partly due to a correlation of reproductive mode and environmental modulation. The development of reproductive structures is often correlated with the ontogeny of lichen thalli, since it has been found that some lichen species use a mixed strategy of early asexual and late sexual reproduction [24]. Besides, macro- and microclimatic variables are also reported to affect reproductive capacity, for example isidia (one type of asexual reproductive structure in lichens) are produced in higher frequency under greater microclimate stress (higher radiation and temperatures, lower humidity) [24]. Some lichenologists found a positive correlation between production of apothecia and microclimatic conditions [25,26], and Seymour et al. [27] showed that lichens more frequently produce sexual structures in hostile environments.

During our studies on the genus *Hypogymnia* (Parmeliaceae) in China, the *Hypogymnia hypotrypa* species group drew our attention as an important case study for assessing the importance of reproductive strategies to delimit species of lichen-forming fungi. The currently recognized species pair includes the sorediate *H*. *hypotrypa* and esorediate *H*. *flavida*. Both taxa are characterized within the genus by a large thallus and yellowish color of the upper surface.

*Parmelia hypotrypa* was initially described by Nylander in 1860 [28] without mention of soredia. Ninety years later another species similar to *P*. *hypotrypa*, but having soredia, was described as *P*. *hypotrypella* [29]. Subsequently, both species were transferred to *Hypogymnia*, as *H*. *hypotrypella* (Asahina) Rassad. [30], and *H*. *hypotrypa* (Nyl.) Rassad. [31]. The syntypes of *P*. *hypotrypa* Nyl. (coll. Hook. &Thoms. nos. 2014–2016) are preserved in H-NYL and BM and subsequently a lectotype was chosen by Awasthi among the syntypes in BM [32]. Both sorediate and non-sorediate thalli were found in parts of its syntypes in H-NYL (No.34197). The non-sorediate thalli were considered as parts of sorediate thalli. Hence, the species *H*. *hypotrypa* was interpreted as being sorediate, and *H*. *hypotrypella* was reduced to synonymy with *H*. *hypotrypa*, and *H*. *flavida* described as a new species to accommodate the non-sorediate specimens [33].

Because species delimitation based on presence or absence of soredia has been shown to be incongruent with phylogenetic relationships in some lichenized fungi, and a correlation between reproductive mode and environmental conditions has also been observed, we reexamined the relationship between *H*. *hypotrypa* and *H*. *flavida*. We reassessed phenotypic characters and generated molecular data to delimit species boundaries in this group. The phenotypic analysis was based on morphological, anatomical, and chemical characters, whereas the molecular data included sequences from the nuclear ribosomal internal transcribed spacer region (ITS) and two protein-coding nuclear markers, *GPD* and *MCM7*. Specifically, our study attempts to assess: (1) whether the presence vs. absence of sorediais diagnostic of two separate lineages in the group, (2) whether presence vs. absence of soredia is related to geography or elevation, and (3) whether other phenotypic characters can be associated with lineages recovered in the phylogenetic analyses.

## Materials and Methods

### Phenotypic study

Over 500 specimens of *H*. *hypotrypa* and *H*. *flavida* collected throughout the species distributions, China (including Taiwan), Japan and Russia, were examined for this study, also including the lectotype of *H*. *hypotrypa* (BM) and holotype of *H*. *flavida* (OSC). No specific permissions were required for these locations/activities. The field studies didn't involve endangered or protected species. A Geographic Information System (GIS) was used to analyze the geographic distribution of *H*. *hypotrypa* and *H*. *flavida*, based on the locality information of examined specimens.

Dissecting (ZeissStemi SV11) and compound (ZeissAxioskop 2 plus) microscopes were used for study of morphology and anatomy. Color test reagents (10% aqueous KOH, saturated aqueous Ca(OCl)_2_, and concentrated alcoholic *p*-phenylenediamine) and standardized thin-layer chromatography (TLC, solvent system C) were used for the identification of secondary metabolites [34,35].

### DNA extraction, PCR amplification, and sequencing

Seventy-four lichen specimens of seven *Hypogymnia* species were selected for DNA extraction based on availability of fresh materials suitable for DNA extraction. The collection information of these specimens is listed in S1 Table, including the latitudes and longitudes of all localities. A total of 62 specimens represented the *Hypogymnia hypotropa* group were collected from a broad geographic range—China (including Taiwan), Japan and Russia—to ensure the range of phenotypic variation. All sequences used in the analyses were newly generated for this study, except sequences of *Arctoparmelia centrifuga*, *Brodoa intestiniformis*, *Letharia* spp. and *Pseudevernia* spp. that were chosen as outgroup and downloaded from GenBank.

The extraction procedure followed a modified CTAB method [36]. The internal transcribed spacer of nuclear ribosomal DNA (nrDNA ITS), and fragments of protein-coding genes GPD (glyceraldehyde 3-phosphate dehydrogenase) and MCM7 (mini-chromosome maintenance proteins) were chosen as the genetic markers. Primers used for the PCR amplifications were listed in Table 1.

**Table 1:** The primers used in the study.

Primer name	Primer sequence (5'→3’)	References
**LR1**	GGTTGGTTTCTTTTCCT	[37]
**ITS1**	TCCGTAGGTGAACCTGCGG	[38]
**Gpd1-LM**	ATTGGCCGCATCGTCTTCCGCAA	[39]
**Gpd2-LM**	CCCACTCGTTGTCGTACCA	[39]
**X_mcm7_F**	CGTACACYTGTGATCGATGTG	[40, 41]
**Mcm7-1348rev**	GAYTTDGCIACICCIGGRTCWCCCAT	[40, 41]

Reactions were carried out in 50 μl reaction volume and the components used were 3 μl total DNA, 1 μl each primer (10 μM), 25 μl 2×Taq MasterMix (CWBIO, China) and 20 μl ddH_2_O. PCR amplifications were carried out in a Biometra T-Gradient thermal cycler, following conditions: initial heating step for 5 min at 95°C, followed by 35 cycles of 30 s at 94°C, 30 s at 56°C for amplifying ITS and GPD sequences or 54°C for amplifying MCM7 sequences, and 1 min 30 s at 72°C, a final extension step of 8 min at 72°C was added, after which the samples were kept at 4°C. Negative controls were prepared for each amplification series. PCR products were purified using gel purification kit (Shanghai Huashun Bioengineering Corporation, China) following the manufacturer’s instructions. PCR products were sequenced using ABI 3730 XL Sequencer by Shanghai BioSune Corporation of China.

### Multiple sequence alignments and data analysis

Sequences were aligned using ClustalW Multiple Alignment [42] in BioEdit 7.2.5 [43]. The alignment files were transformed into phylip format in SeaView 4 [44,45]. Pairwise genetic distances were separated into intraspecific and interspecific parameters and calculated to characterize both intra-and interspecific variation within and between *H*. *hypotrypa* and *H*. *flavida*. Pairwise distances can be viewed as a rough measure for the overall sequence divergence, and an intra-interspecific threshold of ca. 0.015–0.017 substitutions per site has been proposed for species in Parmeliaceae [46]. Pairwise genetic distances were computed for the ITS locus using the general time-reversible model in PAUP* [47] for each nominal taxon individually- *H*. *flavida* and *H*. *hypotrypa*- and all pairwise interspecific comparisons. Genetic distance were then exported from PAUP* and the distribution and mean of pairwise distance were calculated. Pairwise distances between different haplotypes were reported as the number of nucleotide substitutions per site (s/s).

#### Congruence among loci

To test the phylogenetic congruence among loci, well-supported clades in single-gene trees were compared and assessed among individual topologies [48,49]. Each locus was subjected to a randomized accelerated maximum likelihood (RAxML) analysis involving 1000 pseudoreplicates with RAxML-HPC BlackBox 8.2.6 [50] on the Cipres Science Gateway (http://www.phylo.org; [51]). Results were visualized with FigTree 1.4.2 (http://tree.bio.ed.ac.uk/software/figtree/). Individual single locus topologies were visually assessed for well-supported (>75%) conflict compared to the other sampled loci and combined if no conflict was observed [49].

#### Phylogeny of *Hypogymnia hypotrypa* group

Conflicts were not detected in the three single-gene trees, and the three data sets were concatenated. Phylogenetic analyses were performed using RAxML-HPC BlackBox 8.2.6 [50] and MrBayes 3.2.6 [52,53] on the Cipres Science Gateway (http://www.phylo.org; [51]). In the ML analysis, the default GTR + G model was used as the substitution model with 1000 pseudoreplicates. The data was partitioned according to the different genes. For gpd and MCM7 data were also partitioned by codon position. In the Bayesian analysis, the best model for the three single genes had been found in advance with PartitionFinder v1.1.1 [54]. The ITS and the two protein-coding genes data sets were partitioned by the length of sequences and codon position, respectively. Two parallel Markov chain Monte Carlo (MCMC) runs were performed each using 8000,000 generations and sampling every 1,000 steps. A 50% majority rule consensus tree was generated from the combined sampled trees of both runs after discarding the first 25% as burn-in. The tree files were visualized with FigTree 1.4.2 (http://tree.bio.ed.ac.uk/software/figtree/).

### Population genetic analyses and Bayesian clustering

The program SITES [55] was used to assess genetic differentiation and polymorphisms within and between the two traditionally circumscribed taxa in the *H*. *hypotrypa* group, the number of fixed differences, shared polymorphisms and pairwise fixation indices (F_ST_) [56]. To measure genetic differentiation, we used the program DnaSP V5.10.1 [57]. Furthermore, an intra-interspecific threshold of ca. 0.015–0.017 substitutions per site has been proposed for species in Parmeliaceae [46], and comparisons of pairwise genetic distances were made within and between *H*. *flavida* and *H*. *hypotrypa*.

Bayesian clustering implemented in the program STRUCTURE v.2.3.2 [58,59] was used to assign specimens to genetic clusters. All constant nucleotide position characters in the concatenated multi-locus sequence alignment were excluded, and the data matrix for STRUCTURE was comprised of only variable nucleotide position characters (SNPs). Indels and ‘N's were ignored for the purpose of SNP identification. Individual population assignments were inferred for *K* values ranging from 1–5; with 10 replicate runs for each *K* value. Each run consisted of 50,000 burn-in generations, followed by 50,000 iterations using the admixture options.

### Species delimitation analyses

Four species delimitation methods were used to circumscribe species boundaries in the *H*. *hypotrypa* group–“Automatic Barcode Gap Discovery” (ABGD) [60], a Bayesian implementation of the Poisson tree process model (bPTP) [61], the General Mixed Yule Coalescent (GMYC) approach [62,63], and BPP v3.2 [64–66].

For ABGD we used default parameters except for using a Pmax at 0.01 and a relative gap width of 1.5, with the model Jukes-Cantor (JC69). The PTP model is intended for delimiting species in single-locus molecular phylogenies, and provides an objective approach for delimiting putative species boundaries that are consistent with the phylogenetic species criteria. We used the bPTP web server (http://species.h-its.org, [67]) to delimit putative species groups using the ITS topology as the input tree and implementing default settings.

We employed the GMYC approach [62,63] to test whether the data support a scenario supporting all samples in the *H*. *hypotrypa*/*flavida* group as belonging to a single species or not. The GMYC method aims at detecting shifts in branching rates between intra- and interspecific relationships. Within a likelihood framework it uses chronograms to compare a null model under which the whole sample belongs to a single species and hence follows a coalescent process and an alternative general mixed Yule coalescent (GMYC) model. The latter combines equations describing branching patterns within and among lineages. A likelihood ratio test (LRT) is used to evaluate whether the null model can be significantly rejected. If the GMYC model fits the data significantly better than the null model, the threshold T allows estimating the number of species present in the data set. The outgroup samples were excluded from the data set. The GMYC analysis based on the ITS sequences was then run online (http://species.h-its.org/gmyc/), employing a single and multiple threshold methods.

The multispecies coalescent model implemented in the program BPP v3.2 [64–66] was used to assess support for the separation of the sampled *Hypogymnia* species. BPP incorporates coalescent theory and phylogenetic uncertainty into parameter estimation; and the posterior distribution for species delimitation models is sampled using a reversible-jump Markov Chain Monte Carlo (rjMCMC) method. We used the unguided species delimitation algorithm (‘A11’; [68]). This algorithm explores different species delimitation models and different species phylogenies, with fixed specimen assignments to populations. Specimens were assigned to either *H*. *hypotrypa* or *H*. *flavida* based on the conventional phenotype-based descriptions (sorediate vs. esorediate). The program attempts to merge populations into one species, and uses the nearest neighbor interchange (NNI) or subtree pruning and regrafting (SPR) algorithms to change the species tree topology [69]. We used Prior 0, equal probabilities for the labeled histories, to assign probabilities to the models. Rates were allowed to vary among loci (locus rate = 1), and the analyses were set for automatic fine-tune adjustments. Multiple analyses using different combinations of the theta (θ) and tau (τ) priors spanning a range of possible population sizes and divergence times were performed for each genus. The rjMCMC analysis was run for 200,000 generations, sampling every 2 generations discarding the first 10% as burn-in. Each analysis was run twice using a different search algorithm (algorithm 0 or 1) to confirm consistency between runs. Speciation probabilities greater than 0.95 were considered supported species delimitations.

Given that different species delimitation analyses supported different species scenarios for the *H*. *hypotrya* /*flavida* group (see Results), the most likely hypothesis of species boundaries was inferred by comparing marginal likelihoods using Bayes factor delimitation (BFD) test [70]. The optimal species delimitation scenario was evaluated by comparing marginal likelihoods using the BFD framework described previously [70]. We calculated marginal likelihood estimates (MLEs) for three species delimitation scenarios: (i) assigning specimens within the *H*. *hypotrypa*/*flavida* group to two separate species based on traditional, phenotype-based identifications; (ii) lumping all *H*. *hypotrypa*/*flavida* specimens into a single putative lineage; and (iii) assigning specimens within the *H*. *hypotrypa*/*flavida* group to two separate candidate species-level lineages based on the results of the PTP analysis (see Results). All other sampled *Hypogymnia* species were assigned species-level membership based on morphological identifications.

For each of the three species delimitation scenarios we reconstructed a species tree using *BEAST v1.8.3 [71]. Substitution models for each of the three loci were chosen using PartitionFinder [54], as described above. We selected a birth—death model for the species tree prior; the population size model was set to piecewise linear and constant root. *BEAST analyses were performed using 20,000,000 generations, sampling every 1000 generations, and the first 25% of each run was discarded as burn-in. MLEs were estimated using the stepping-stone method [72], with 100 path steps, a chain length of 100,000 generations and likelihoods saved every 100 generations. Bayes factors were then calculated as described by Grummer et al. [70], with 2lnBf >10 being considered as ‘decisive’ support for a hypothesis.

## Results

### Phenotypic studies

**Fig 1:**
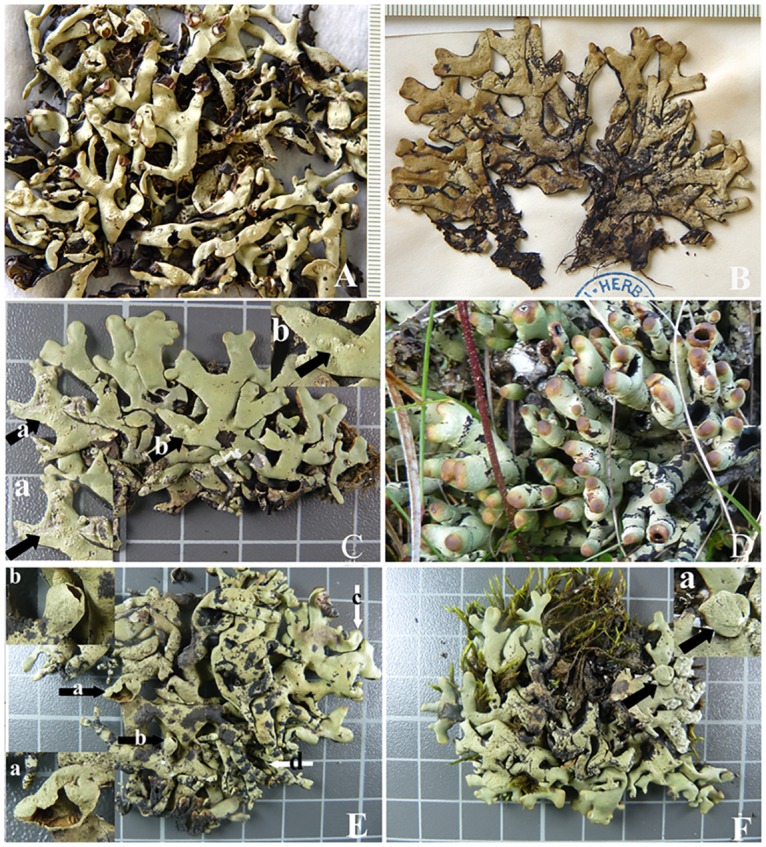
Morphology of the *Hypogymnia hypotrypa* group. A. Holotype of *H*. *flavida* (OSC). B. Lectotype of *H*. *hypotrypa* (BM). C. Soredia of *H*. *hypotrypa*, X. L. Wei W11135; a, b: soredia present only along the cracks of upper cortex. D. Fingerlike lobes of *H*. *flavida*, X. L. Wei W11188 (HMAS-L). E. *H*. *flavida*, X. L. Wei W11231 (HMAS-L); a, b: apothecia; c: wide lobes; d: fingerlike lobes. F. *H*. *hypotrypa*, wide lobes, X. L. Wei W11129 (HMAS-L); a: apothecia. A-F: scale in cm.

All sampled specimens from the *H*. *hypotrypa* group (*H*. *hypotrypa* and *H*. *flavida*) were identical in the anatomical structure and chemical substances, both of which developed internally heteromerous thalli: prosoplectenchymatous upper cortex, photobiont layer, medulla and prosoplectenchymatous lower cortex with similar thicknesses. However, in rare instances some lobes tip of *H*. *flavida* lacked obvious dorsoventrality (Fig 1D), resulting in two upper cortex layers and two algal layers. In chemistry, the *H*. *hypotrypa* group constantly contained usnic acid, physodalic acid, and protocetraric acid; some also contained 3-hydroxyphysodic acid. The only apparent phenotypic differences between *H*. *flavida* and *H*. *hypotrypa* were in regards to lobe morphology and presence of soredia. Although soredia were present in all *H*. *hypotrypa* specimens, in many cases, the soredia were distributed along the cracks of the upper surface and hence could easily be overlooked (Fig 1C). Compared with *H*. *hypotrypa*, *H*. *flavida* had a broader range of variation in lobe morphology. In addition to the broad and richly branched lobes typical of *H*. *hypotrypa* (Fig 1A–1E), the lobes of *H*. *flavida* were occasionally found to be fingerlike and sparsely branched (Fig 1D). Production of apothecia was observed in both *H*. *hypotrypa* and *H*. *flavida* (Fig 1E and 1F).

### Geographic distribution

**Fig 2:**
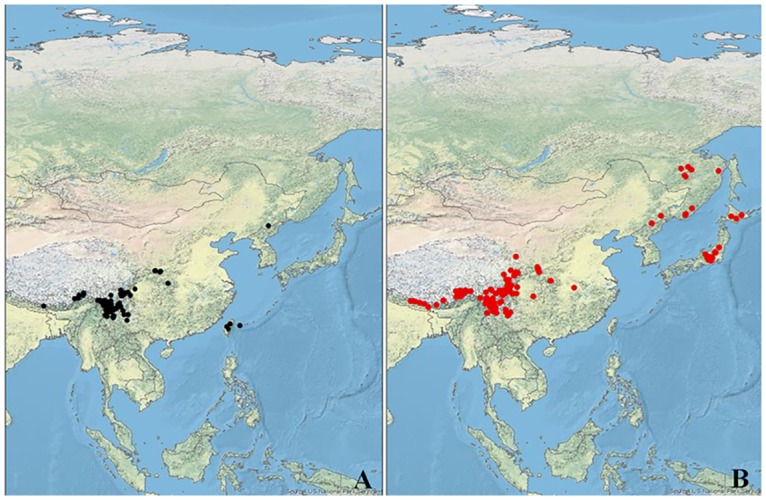
The geographic distribution of examined specimens of the two examined *Hypogymnia* species. A. *Hypogymnia flavida*; B. *Hypogymnia hypotrypa*. Basemap source: U.S. National Park Service (NPS) Natural Earth physical map at 1.24 km per pixel.

Both *H*. *hypotrypa* and *H*. *flavida* usually grow in alpine to montane habitats in eastern Asia, although each species is known to occur across a broader altitudinal range. Based on the analysis of over 500 specimens in our study and in agreement with previous results [33], *H*. *hypotrypa* has a broader geographic distribution and wider altitudinal range. *Hypogymnia flavida* can be found between 2150 m to 4300 m, while *H*. *hypotrypa* is found at an altitude between 65 m to 4300 m. We confirm the occurrence of *H*. *flavida* in China (including Taiwan), and *H*. *hypotrypa* in China, Japan and Russia (Fig 2). *Hypogymnia hypotrypa* has also been reported from Taiwan and North Korea [33,73– 77], but we have not seen this material and thus cannot confirm the identity of these collections.

### Molecular data

Eighty nrDNA ITS sequences (508 aligned nucleotide position characters [bp]), 68 GPD sequences (757 bp), and 47 MCM7 sequences (594 bp) were used in the analyses, among which 187 DNA sequences were newly generated for this study. The complete, three-locus matrix was comprised of 80 specimens and was comprised of 1859 aligned nucleotide position characters, among which 1580 were constant.

### Genetic differentiation and Bayesian clustering

**Fig 3:**
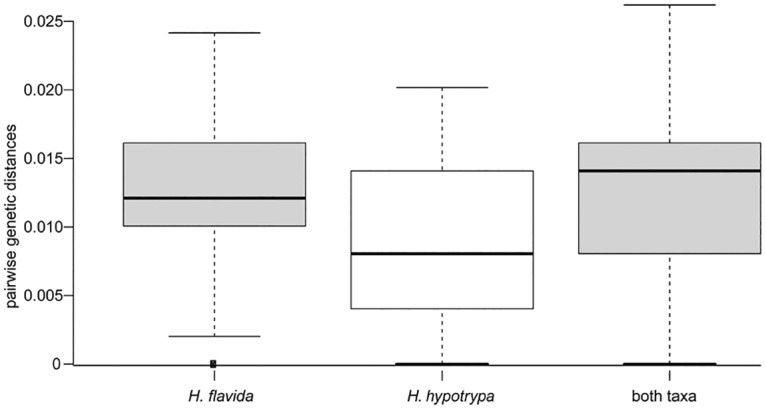
Box plot representation of the intra- and interspecific pairwise genetic distances for the *Hypogymnia hypotrypa* group. In each box plot, the box shows the interquartile range (IQR) of the data. The IQR is defined as the difference between the 75th percentile and the 25th percentile. The whiskers represent variability outside the upper and lower quartiles. The solid line through the box represents the median pairwise genetic distance.

No fixed differences in nucleotide positions were observed between *H*. *hypotrypa* and *H*. *flavida* in any of the three sampled loci (Table 2). *F*_ST_ indices were calculated to assess the degree of genetic isolation within *H*. *hypotrypa* group, which can vary from 0 (complete panmixis) to 1 (complete isolation between populations). In our study, the values of *F*_ST_ were relatively low, ranging from 0.035 to 0.276. The shared polymorphisms revealed 5–10 nucleotide shared by *H*. *hypotrypa* and *H*. *flavida*. The range of genetic distances for *H*. *hypotrypa* and *H*. *flavida* were summarized in Fig 3. For both species, most of intraspecific pairwise comparisons fell below the proposed intra-interspecific threshold 0.015–0.017 substitutions per site. The range of genetic distances was similar when mixing the samples of *H*. *flavida* and *H*. *hypotrypa* together as one species, although this yield a limited number distances above this threshold (ca. 0.026 s/s).

**Table 2:** Analysis of DNA polymorphisms and Fst values for a comparison of *H*. *flavida* and *H*. *hypotrypa*.

Method	Gene marker	Fixed differences	Shared polymorphisms	*F*_ST_
SITES	ITS	0	9	0.276
	GPD	0	10	0.102
	MCM7	0	8	0.035
DNASP	ITS	0	8	
	GPD	0	10	
	MCM7	0	5	

Note: Fixed nucleotide position characters and shared polymorphisms were identified for each sampled loci—ITS, GPD, and MCM7—using the programs SITES and DnaSP. *F*_st_ values were calculated for each using the program SITES.

**Fig 4:**
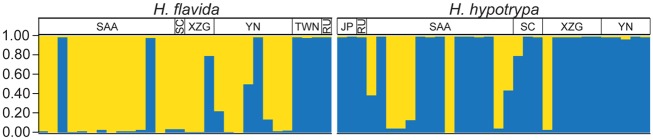
Results from a Bayesian genetic clustering analysis of the *Hypogymnia hypotrypa* group. Individual population assignments were inferred using a STRUCTURE analysis of single nucleotide polymorphisms from multi-locus sequence data from specimens identified as *H*. *flavida* and *H*. *hypotrypa* under a model assuming two genetic groups. The horizontal axis gives specimen numbers. The vertical axis represents the inferred proportion of each individual’s genome assigned to a genetic cluster, with assignment probability into the two different genetic clusters depicted with distinct colors–'cluster 1' shown in yellow and 'cluster 2' in blue. Specimens within each taxon are clustered by geographic region (SAA = Shaanxi; YN = Yunnan; XZG = Tibet; SC = Sichuan; TWN = Taiwan; JP = Japan; and RU = Russia). Population assignments for each specimen are reported in S1 Table.

Results from the Bayesian clustering analysis of the *H*. *hypotrypa*/*flavida* group performed under the assumption of two distinct populations are shown in Fig 4. Specimens representing each traditionally circumscribed species were recovered in two distinct genetic clusters–'cluster 1' and 'cluster 2', with approximately 10% of samples specimens with evidence of admixed genomes. However, the majority of *H*. *flavida* specimens were assigned membership to 'cluster 1', while those identified as *H*. *hypotrypa* were assigned membership to 'cluster 2' (Fig 4). The information of samples from different localities assigning to 'cluster 1' and 'cluster 2' is seen in S1 Table.

### Phylogenetic analyses

**Fig 5:**
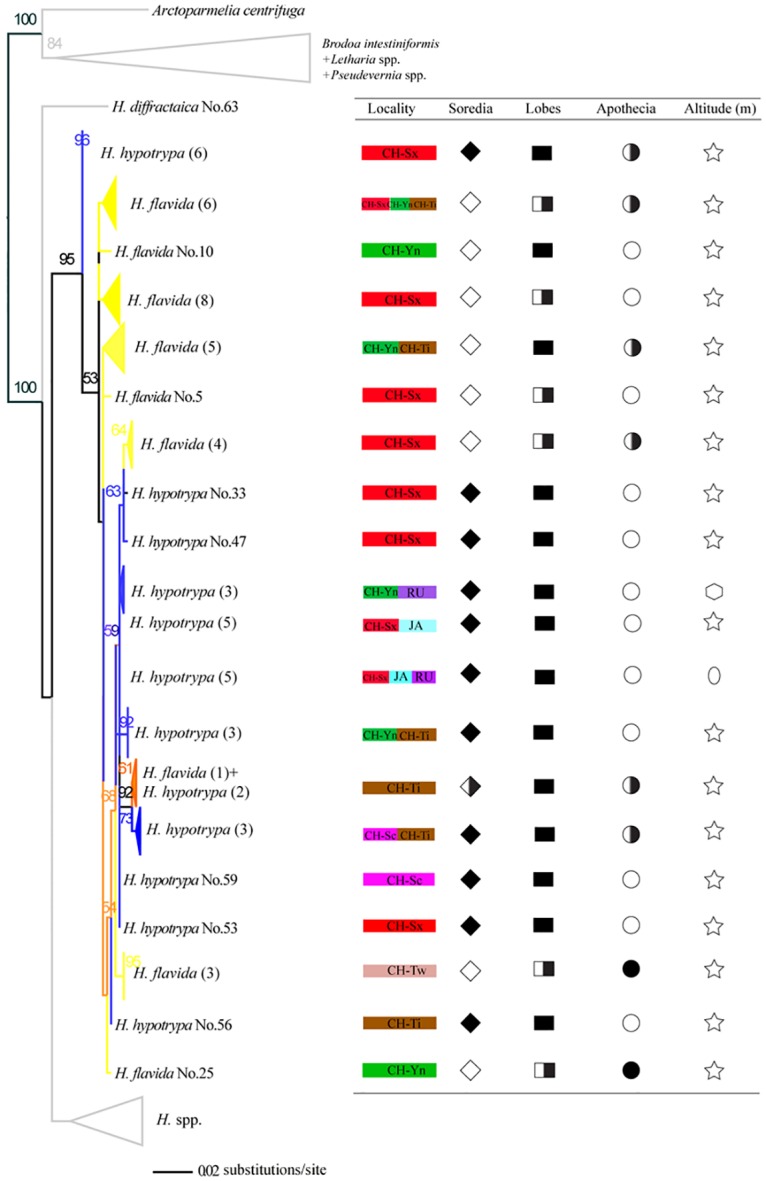
Cartoon topology of ML phylogenetic tree based on nrDNA ITS. The numbers in each node represents bootstrap support value, and the numbers lower than 50 were not shown. The number of each sample (i.e. No.) is listed in S1 Table, while the number in the bracket indicates that the amount of samples corresponding to the same species in the same sub-branch. Three colors are included in the branches (blue = *H*. *hypotrypa*, yellow = *H*. *flavida*, orange = both *H*. *hypotrypa* and *H*. *flavida*). The branches of other species of *Hypogymnia* and outgroup are shown in gray color. Right table indicates the collection locality information and main morphological characters delimiting *H*. *hypotrypa* and *H*. *flavida*. Red = Shaanxi Province of China (CH-Sx), green = Yunnan Province of China (CH-Yn), purple-red = Sichuan Province of China (CH-Sc), brown = Tibet of China (CH-Ti), pink = Taiwan of China (CH-Tw), pale blue = Japan (JA), purple = Russia (RU). Soredia is indicated by ◇ (◇ = absence, ◆ = presence, half filled ◇ = absence sometimes), lobes by ■ (■ = wide, half filled □ = both wide and fingerlike), and apothecia by ○ (○ = absence, ● = presence, half filled ○ = absence sometimes). ☆, shows all the samples in the sub-branch distribute at the high altitude (more than 2000 meters high); hexagon, part at the middle altitude (about 500 m); oval, part at low altitude (about 50 m).

**Fig 6:**
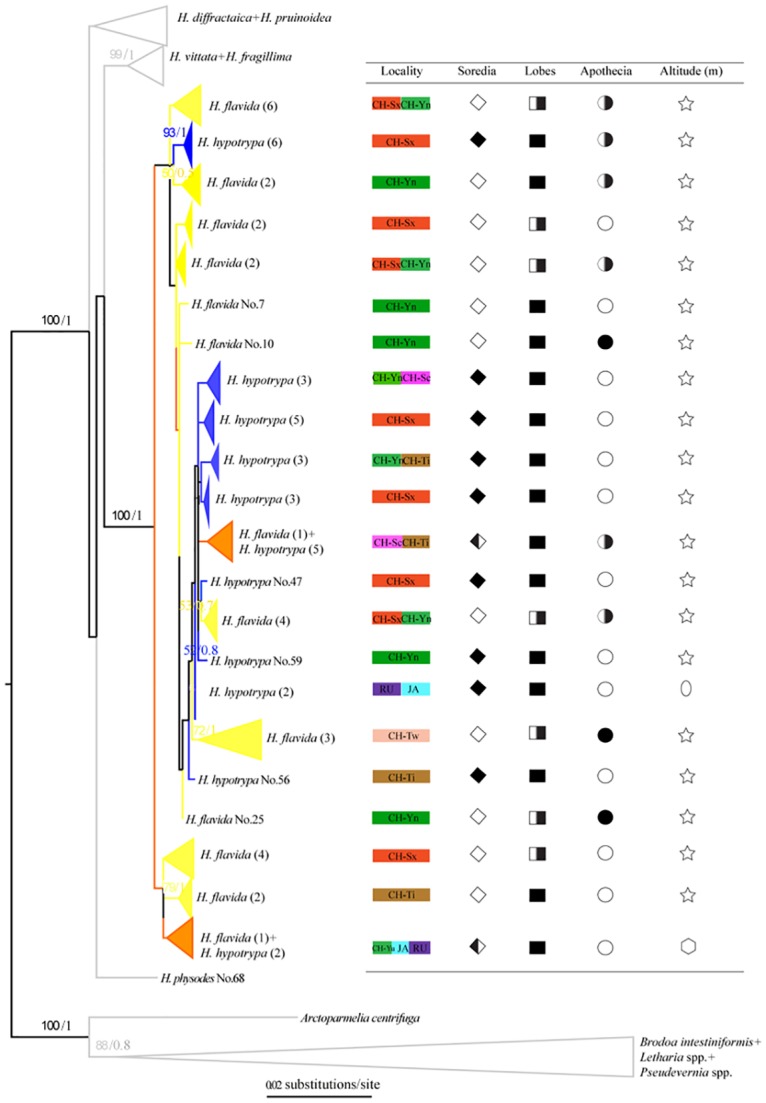
Cartoon topology of ML phylogenetic tree based on three loci. The numbers in each node represents bootstrap support and posterior probability values based on Bayesian analysis, and the numbers lower than 50 (BS) and 0.5 (PP) were not shown. The number of each sample (i.e. No.) is listed in S1 Table, while the number in the bracket indicates that the amount of samples corresponding to the same species in the same sub-branch. Three colors are included in the branches (blue = *H*. *hypotrypa*, yellow = *H*. *flavida*, orange = both *H*. *hypotrypa* and *H*. *flavida*). The branches of other species of *Hypogymnia* and outgroup are shown in gray color. Right table indicates the collection locality information and main morphological characters delimiting *H*. *hypotrypa* and *H*. *flavida*. Red = Shaanxi Province of China (CH-Sx), green = Yunnan Province of China (CH-Yn), purple-red = Sichuan Province of China (CH-Sc), brown = Tibet of China (CH-Ti), pink = Taiwan of China (CH-Tw), pale blue = Japan (JA), purple = Russia (RU). Soredia is indicated by ◇ (◇ = absence, ◆ = presence, half filled ◇ = absence sometimes), lobes by ■ (■ = wide, half filled □ = both wide and fingerlike), and apothecia by ○ (○ = absence, ● = presence, half filled ○ = absence sometimes). ☆, shows all the samples in the sub-branch distribute at the high altitude (more than 2000 meters high); hexagon, part at the middle altitude (about 500 m); oval, part at low altitude (about 50 m).

Single-locus maximum likelihood (ML) topologies and ML and Bayesian trees inferred from the concatenated, 3-locus data set (1859bp) are shown in S1–S5 Figs. In order to clearly depict relationships among *H*. *hypotrypa* and *H*. *flavida* specimens, cartoon topologies of the ITS and concatenated matrix are reported (Figs 5 and 6). Because the topology of ML and Bayesian trees are highly similar, the posterior probability values above 0.5 are noted directly after the bootstrap values at the nodes of the ML tree (Fig 6). The *H*. *hypotrypa* group formed a well-supported clade (BS = 90, 100 and PP = 1) and was comprised of closely related specimens distinct from other sampled species of *Hypogymnia* species (Figs 5 and 6).

Within the *H*. *hypotrypa*/*flavida* clade, several samples of *H*. *hypotrypa* (blue branches) or *H*. *flavida* (Figs 5 and 6; yellow branches) clustered into small sub-branches, then intermixed each other. In some cases, samples of *H*. *hypotrypa* and *H*. *flavida* clustered together, forming separated sub-clades (orange branches). No formation of two well defined separated bigger clades corresponds to *H*. *hypotrypa* and *H*. *flavida*. No obvious relationship between clades andlarge-scale geographic distribution were seen, although we found small-scale geographic differentiation. For example, some samples from Shaanxi Province of China (CH-Sx, highlighted in red) often formed separated sub-clades. Samples from Japan (JA, highlighted in pale blue) and Russia (RU, highlighted in purple) had a closer relationships to each other than either of those areas to populations from China. Most notably, *H*. *flavida* from Taiwan (CH-Tw, highlighted in pink) always intermixed with *H*. *hypotrypa*, showing a close relationship with *H*. *hypotrypa* from Tibet (CH-Ti, highlighted in brown).

Corresponding to the topology of *H*. *hypotrypa* and *H*. *flavida* in the phylogenetic trees (Figs 5 and 6), presence/absence of soredia didn't correspond to monophyletic groups in any phylogenetic reconstructions, except that some separation was seen in portions of the tree. Differences in lobe morphology and presence of apothecia did not correspond with the traditional circumscriptions of *H*. *hypotrypa* and *H*. *flavida*, or any monophyletic clade in our molecular phylogenies. Monophyletic groups corresponding to ecological or geographic regions were not observed for specimens representing the *H*. *hypotrypa* group.

### Species delimitation analyses

The ABGD analysis based on nrDNA ITS provided evidence supporting one species delimitation scenario, e.g. all specimens within the *H*. *hypotrypa*/*flavida* group are inferred as conspecific (P = 0.0021–0.01). The ABGD circumscription of all specimens within the *H*. *hypotrypa*/*flavida* group as a conspecific OTU was concordant to the lack of deep, well-supported phylogenetic substructure within this clade.

The tree-based bPTP analysis suggested two species (S6 Fig), 'species 1' included samples *H*. *hypotrypa* Nos 34–36, 40–41, 49 (CH-Sx), and 'species 2' comprised of all remaining samples of *H*. *hypotrypa* and *H*. *flavida*. Although the bPTP analysis circumscribed two species, the posterior probabilities supporting these species was quite low, 0.1 and 0.0 values for 'species 1' and 'species 2', respectively. Furthermore, the six samples of *H*. *hypotrypa* in 'species 1' were not supported as a monophyletic clade in the other single gene topologies (S2–S4 Figs).

In the GMYC analyses using the single and multiple threshold methods, the GMYC model was not significantly better than the null model of uniform (coalescent) branching rates. The likelihood ratio for the single threshold method analyzing on the ingroup *(H*. *flavida* and *H*. *hypotrypa*) was 1.3, and three or four clusters within *H*. *hypotrypa* group were included (S7 Fig). More than 10 clusters were shown when the multiple threshold method was performed, which seems rather unreasonable because most of time one cluster was only composed of two or three samples. It has previously been suggested that the single-threshold model outperforms the multiple-threshold version [78], and results from the multiple threshold GMYC analysis were not considered further.

In contrast to the ITS-based species delimitation analyses, the multispecies coalescent species delimitation method BPP provided strong support (posterior probability = 1.0) for the recognition of *H*. *flavida* and *H*. *hypotrypa* as distinct species-level lineages. Additionally, the BFD test provided decisive support for the model circumscribing *H*. *flavida* and *H*. *hypotrypa* as distinct species, over a species model of conspecificity for this group or the species delimitation scenario inferred using bPTP (Table 3).

**Table 3:** Marginal likelihood and Bayes factor values for alternative species delimitation scenarios.

Species delimitation scenario	Ln (marginal likelihood)	2ln (Bayes factor)
*H*_1_—H. flavida/H. hypotrypa split	**-4512.4873**	N/A
*H*_2_ H. flavida/H. hypotrypa merged	-4545.6138	66.253
*H*_3_PTP	-4534.3863	43.798

Note: Marginal-likelihood estimates and Bayes factor testing results (2lnBf)BF = 2 x (model1—model2); The model receiving the best marginal-likelihood score for each estimation method is indicated by a 2lnBf score = N/A, and its associated marginal likelihood is in bold.

## Discussion

In this study we used an integrative approach to investigate species boundaries between two closely related lichen-forming fungal species in the genus *Hypogymnia*, *H*. *flavida* and *H*. *hypotrypa*. The production of vegetative reproductive propagules in *H*. *hypotrypa*, differences in lobe morphology, and variation in geographic distributions have traditionally separated species in the *H*. *hypotrypa* group. Our morphological analyses of over 500 specimens supported the previous observations that *H*. *hypotrypa* and *H*. *flavida* differ in the presence or absence of soredia, wide or narrow lobes, and the former had a broader geographic distribution. However, in this study, analyses based on the DNA sequences data failed to provide a consensus on species boundaries in *H*. *hypotrypa*/*flavida* group.

The three species delimitation analyses based on ITS sequence data alone—ABGD, bPTP, and GMYC—indicated multiple scenarios of species boundaries in the *H*. *hypotrypa*/*flavida* group: one being that all members of this group are conspecific (ABGD), while bPTP and GMYC support multiple species-level lineages within this group. However, candidate species circumscribed using bPTP and GMYC did not correspond with the traditional diagnostic character of the presence or absence of soredia.

Although both bPTP and GMYC delimited multiple candidate species within the *H*. *hypotrypa*/*flavida* clade, the supporting evidence was not particularly robust. The Bayesian implementation of PTP provided only weak statistical support for the two species delimited in this group, with posterior probabilities << 0.5 (see Results), and the GMYC model was not significantly better than the null model of uniform branching rates. Similar to the results of the ABGD analysis which suggested *H*. *hypotrypa* and *H*. *flavida* to be conspecific, *F*_ST_ values between *H*. *hypotrypa* and *H*. *flavida* were relatively low, e.g. from 0.035 to 0.276, suggesting little isolation between the two nominal species. Additionally, there were 10 shared polymorphisms at most, supporting the hypothesis that the nominal taxa *H*. *hypotrypa* and *H*. *flavida* do not form two distinct evolutionarily independent lineages.

In contrast, the coalescent-based species validation method BPP (see Results) and BFD tests (Table 3) provided decisive evidence supported the recognition of *H*. *flavida* and *H*. *hypotrypa* as distinct separate species. If the independence of these nominal taxa is legitimate, it is not tracked by the ITS marker, the formal barcoding marker for fungi [79], suggesting a recent diversification history for this clade. This result highlights a potential limitation of using single-locus datasets and phylogenetic species recognition criteria for groups with recent diversification histories and incomplete sorting among lineages [80]. However, the relatively high intraspecific genetic distances in both *H*. *flavida* and *H*. *hypotrypa*, with some pairwise comparisons exceeding the proposed threshold for species in Parmeliaceae [46], suggest the potential for more complex species delimitation scenarios. Recently, phylogenomic data has shown promise in resolving relationships in closely related lichen-forming fungal species groups with recent divergence histories [81], and we anticipate that genome-scale data will provide important insight and resolution into relationships in the *H*. *hypotrypa*/*flavida* group.

Species in the *H*. *hypotrypa* group were not recovered as monophyletic in phylogenetic analyses of multilocus DNA sequence data. Additional species delimitation analyses, genetic clustering, and comparisons of genetic differentiation indicated multiple possible scenarios of species boundaries in the *H*. *hypotrypa* group, e.g. conspecificity or multiple independent species. This raises the question, what are the existing limitations in delimitating species boundaries using molecular sequence data and phylogenetic analyses and what are the limitations of traditionally diagnostic phenotypic characters?

In regards to our initial question about the taxonomic utility of the presence or absence of soredia, our data suggest that differences in reproductive strategies may not correspond to species boundaries with high fidelity (Fig 4; STRUCTURE). In some groups, molecular data suggested that the sorediate and non-sorediate taxa were conspecific, and the sorediate populations usually have a larger range (e.g., *Usnea antarctica* morph of *U*. *aurantiaco-atra*) [23]. Phenotypically, *H*. *hypotrypa* and *H*. *flavida* differ in the presence or absence of soredia, and they vary in production of wide or narrow lobes. Furthermore, *H*. *hypotrypa* has a broader geographic distribution. The geographical ranges of *Hypogymnia hypotrypa* and *H*. *flavida*, however, are more complex with esorediate morphs absent from Russia and Japan and sorediate morphs absent from Taiwan of China. While the former agrees with other studies, the absence of sorediate morphs from Taiwan is difficult to interpret and may be due to the fact that populations belonged to different species. Although *H*. *hypotrypa* had not been confirmed in Taiwan, our data indicate that *H*. *flavida* fromTaiwan has a closer relationship to *H*. *hypotrypa* than to specimens identifiable *as H*. *flavida* from other localities (Figs 5 and 6, S1–S5 Figs). This can be interpreted in several ways: (1) *H*. *flavida* of Taiwan represents *intermediates by introgression* between *H*. *hypotrypa* and *H*. *flavida*, (2) *H*. *flavida* from Taiwan is close to the ancestral state at the time of divergence of sorediate and esorediate lineages, but is currently reproductively isolated from both *H*. *hypotrypa* and continental *H*. *flavida*, or (3) the pattern represents a random deviation in an otherwise panmictic species complex. For any of these three scenarios, one could conclude that *H*. *flavida* is conspecific with *H*. *hypotrypa*. But both scenarios 1 or 2 are also compatible with a taxonomy that accepts two or more species, using a phylogenetic species concept.

However, this scenario contradicts the results of the BPP species validation analysis and BFD test, which support the traditional recognition of species based on the presence or absence of soredia to delimit the *H*. *hypotrypa* group. The presence or absence of soredia may generally correspond to distinct evolutionary lineages, e.g., *H*. *hypotrypa* and *H*. *flavida*, but may not be a consistent diagnostic feature (see Fig 4). The misspecification of individuals in coalescent-based species delimitation analyses, such as BPP, is not well understood. The strong support in BPP and BFD tests may reflect the general pattern of the presence or absence of soredia in each lineage, rather than an exclusive pattern in each group.

The influence of reproductive mode on distributional ranges of lichens is currently poorly understood [82]. *Hypogymnia* species with soredia tend to have broader transcontinental ranges than esorediate species [83]. Poelt [84] showed that sorediate populations are generally expected to have higher potential for long-distance dispersal and hence often have larger distributional ranges. The elevation range of the esorediate taxon *H*. *flavida* (2150–4300 m) is about half that of the sorediate form *H*. *hypotrypa* (65–4300 m), which occurs in high montane to subalpine or alpine habitats. Note that this vertical difference is exactly analogous to the broader distribution observed for sorediate counterparts to fertile species. Higher altitude habitats may be correlated with harsher environmental conditions. Ecological stress, including biotic and abiotic factors, as important correlation factors contributing to genomic and phenomic diversity in nature, and has been shown to bepositively correlated with increased sexuality (by means of meiospores) in lichens and soil microfungi [27,85,86]. Because *H*. *flavida* grows exclusively at higher elevations, it would be not surprising having some adaptive phenotype under this ecological stress, such as narrower finger-like lobes, differing from the most common wide lobes of both species, and depending on sexual reproduction but not on vegetative reproduction.

Previous studies of other lichen genera have suggested that some sorediate and esorediate populations likely belong to a single species [14,20,22,23, 87–89]. Our data from the *H*. *hypotrypa* group suggest a more complex relationship between esorediate and sorediate populations, including the presence of reproductively uniform species being closely related to lineages exhibiting different reproductive modes [90] or the presence of several sorediate populations each representing distinct lineages [91,92]. Despite the fact that in the majority of cases studied using molecular data sorediate and esorediate populations were found to represent variations within one species, no conclusions can be drawn *a priori*. The lack of a generalizable pattern in the taxonomic utility of differences in reproductive strategies demonstrates that each case requires careful consideration. The genus *Hypogymnia* is a prime example since it includes distinct lineages characterized by the morphology of soralia [93–95].

## Supporting Information

S1 Fig The RAxML tree based on nrDNA ITS sequences. The numbers in each node represents bootstrap support value, and the numbers lower than 50 were not shown. The samples marked with ‘○’ were downloaded from GenBank, and others were newly generated for this analysis. The number of each sample is listed in S1 Table.
(TIF)

S2 Fig The RAxML tree based on GPD sequences. The numbers in each node represents bootstrap support value, and the numbers lower than 50 were not shown. The samples marked with ‘○’ were downloaded from GenBank, and others were newly generated for this analysis. The number of each sample is listed in S1 Table.
(TIF)

S3 Fig The RAxML tree based on MCM7 sequences. The numbers in each node represents bootstrap support value, and the numbers lower than 50 were not shown. The samples marked with ‘○’ were downloaded from GenBank, and other were newly generated for this analysis. The number of each sample is listed in S1 Table.
(TIF)

S4 Fig The RAxML tree based on 3-loci concatenated sequences. The numbers in each node represents bootstrap support value, and the numbers lower than 50 were not shown. The samples marked with ‘○’ were downloaded from GenBank, and others were newly generated for this analysis. The number of each sample is listed in S1 Table.
(TIF)

S5 Fig The Bayesian tree based on a concatenated 3-locus data matrix. The numbers in each node represents posterior probability value, and the numbers lower than 0.5 were not shown. The samples marked with ‘○’ were downloaded from GenBank, and others were newly generated for this analysis. The number of each sample is listed in S1 Table.
(TIF)

S6 Fig The Maximum likelihood solution generated by bPTP (a Bayesian implementation of the Poisson tree process model) based on ITS. The numbers in each node represents support value. The red color indicates the PTP suggested species, while blue for uncertain. Two main groups suggested here were corresponding to two species (Sp.1 & Sp.2).
(TIF)

S7 Fig The dichotomous chronogram generated by GMYC based on ITS using single threshold model with exclusion of outgroups. The separated species or populations were indicated by the black lines, while the red line showed the individuals within each species or populations.
(TIF)

S1 Table Specimens used for DNA extraction and sequences used in this study. (DOC)
