# Supplementary figures and images for: Limitations of Species Delimitation Based on Phylogenetic Analyses: A Case Study in the Hypogymnia hypotrypa Group (Parmeliaceae, Ascomycota)
Source: PLoS One. 2016 Nov 9;11(11):e0163664. doi: 10.1371/journal.pone.0163664 (PMC5102465; doi:10.1371/journal.pone.0163664)

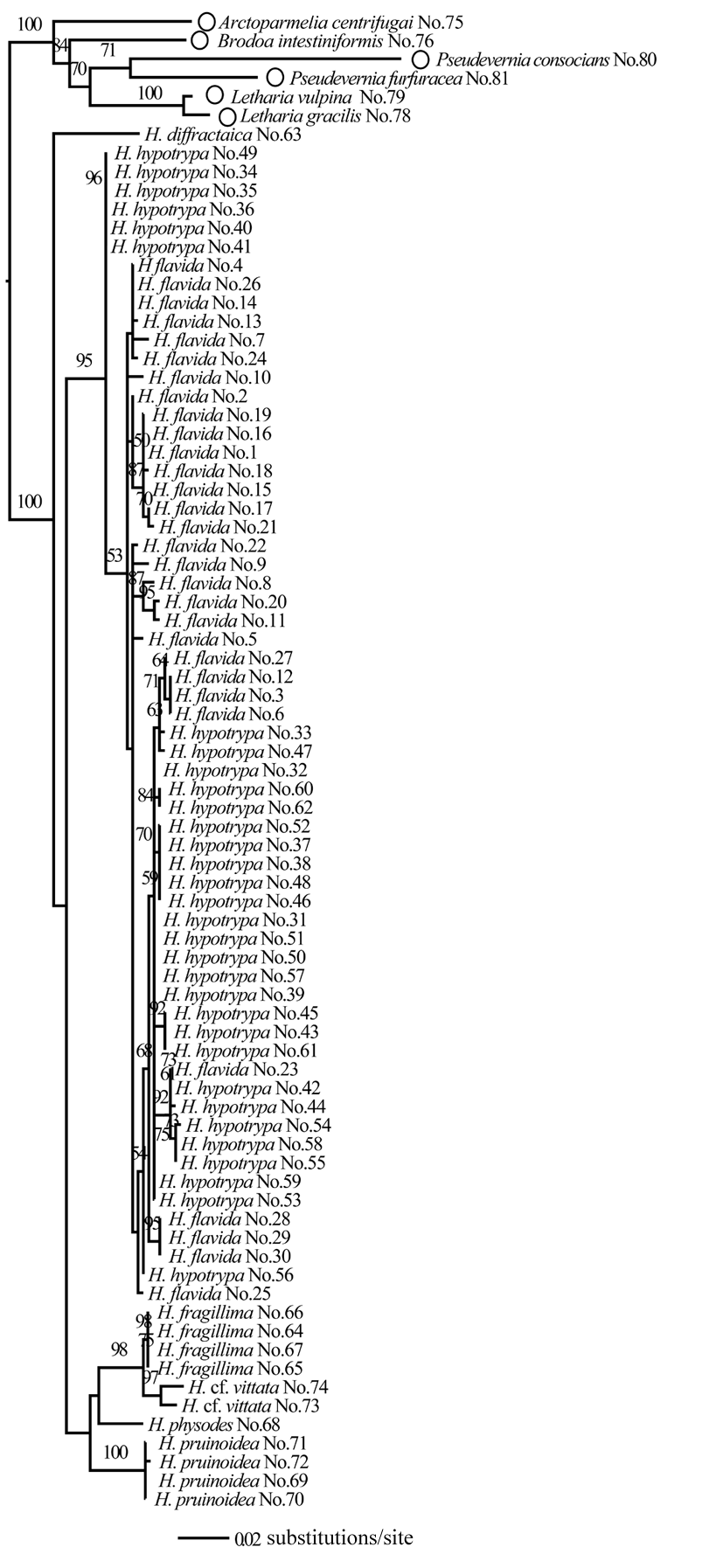

Supplement: S1 Fig — The numbers in each node represents bootstrap support value, and the numbers lower than 50 were not shown. The samples marked with ‘○’ were downloaded from GenBank, and others were newly generated for this analysis. The number of each sample is listed in S1 Table. (TIF) [file pone.0163664.s001.tif]

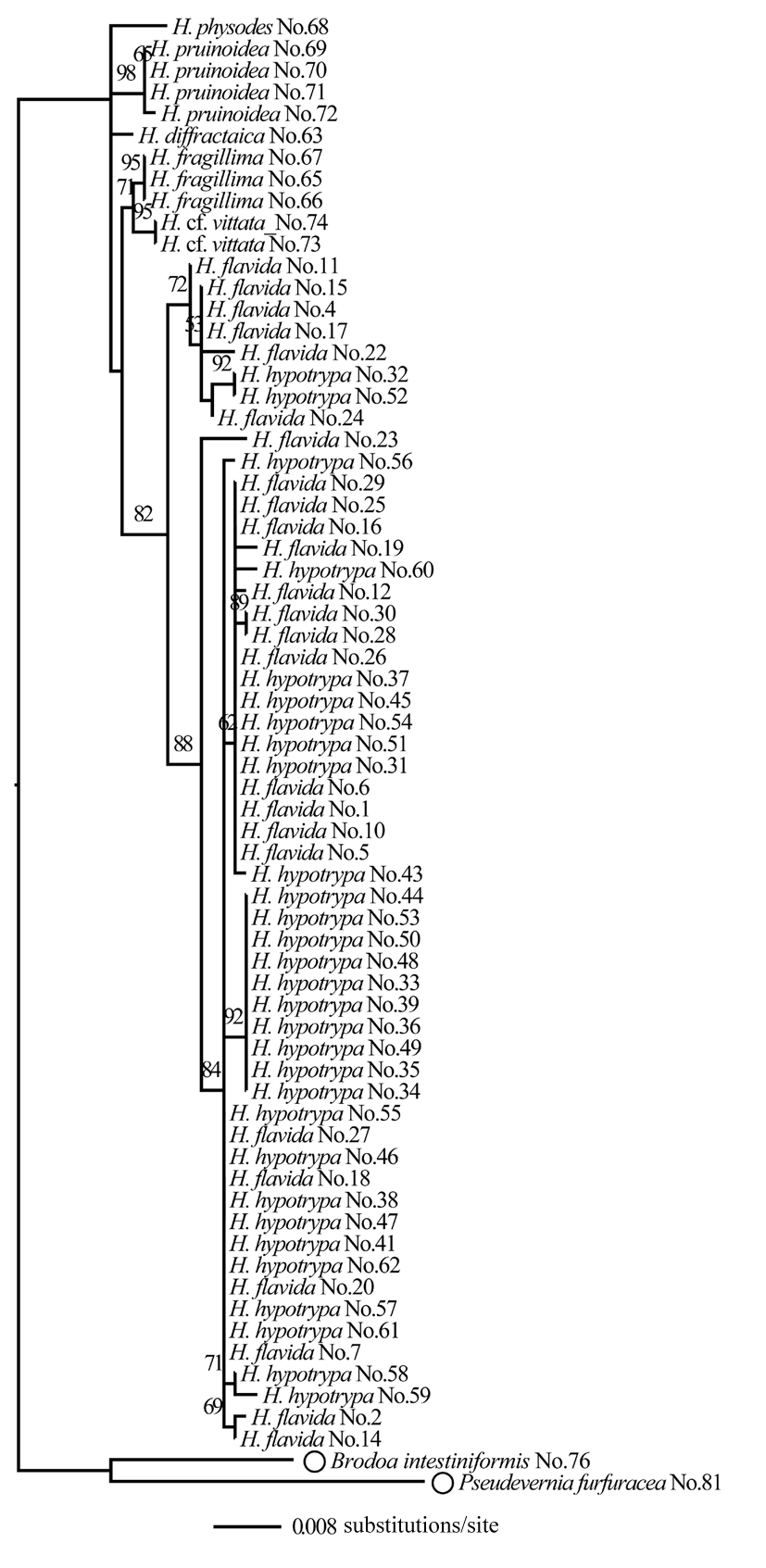

Supplement: S2 Fig — The numbers in each node represents bootstrap support value, and the numbers lower than 50 were not shown. The samples marked with ‘○’ were downloaded from GenBank, and others were newly generated for this analysis. The number of each sample is listed in S1 Table. (TIF) [file pone.0163664.s002.tif]

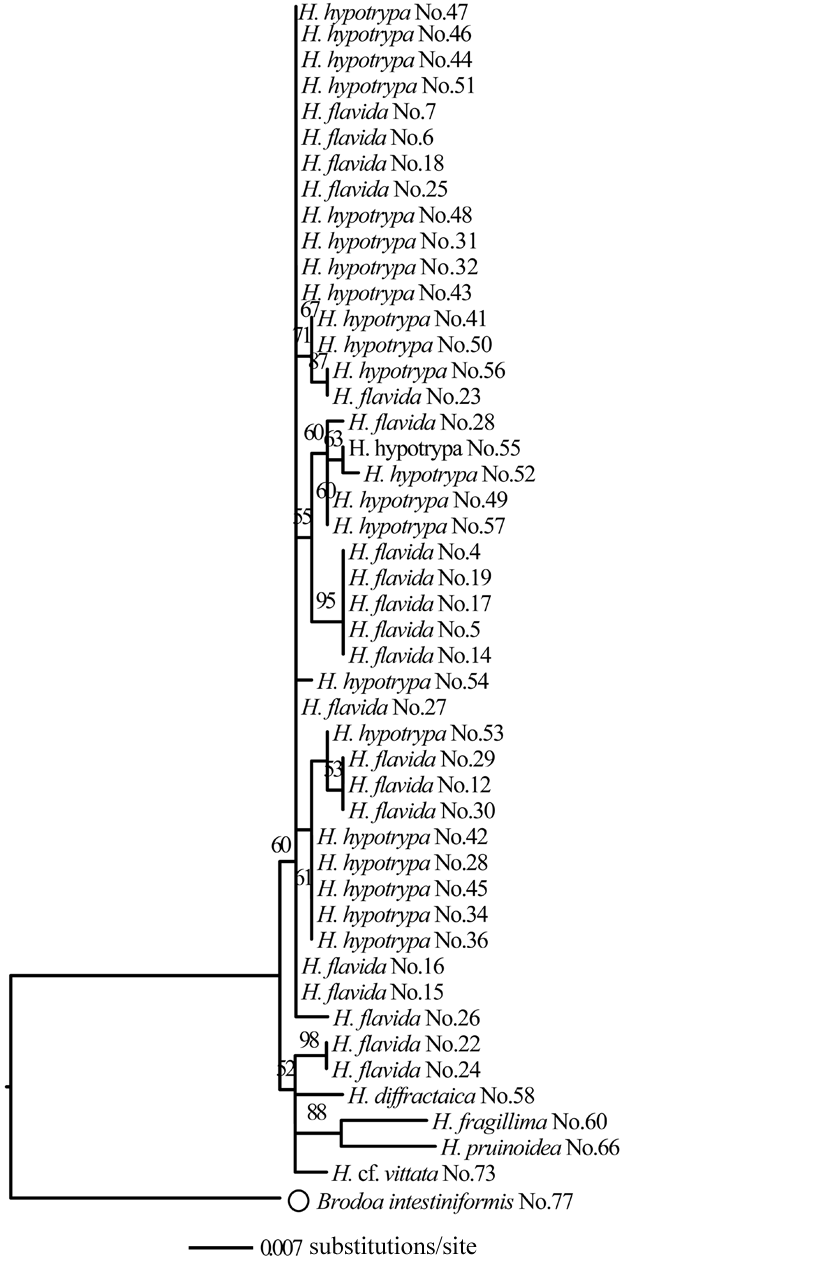

Supplement: S3 Fig — The numbers in each node represents bootstrap support value, and the numbers lower than 50 were not shown. The samples marked with ‘○’ were downloaded from GenBank, and other were newly generated for this analysis. The number of each sample is listed in S1 Table. (TIF) [file pone.0163664.s003.tif]

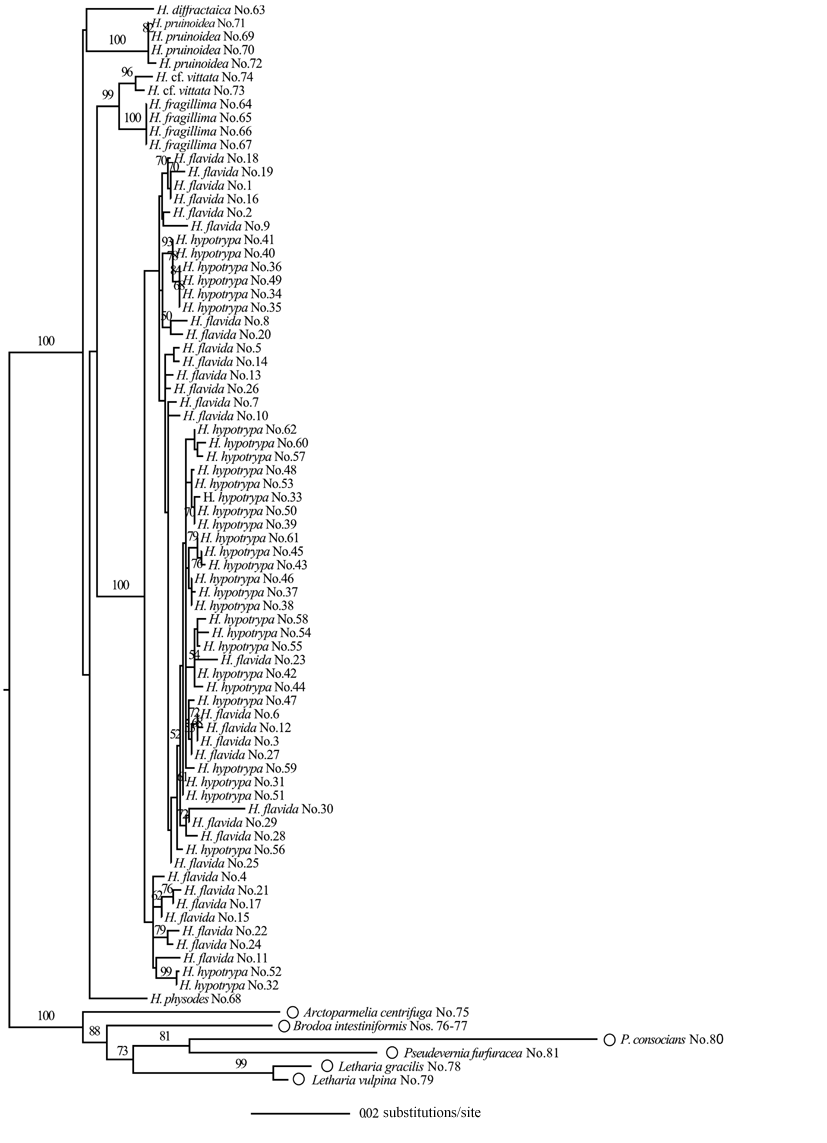

Supplement: S4 Fig — The numbers in each node represents bootstrap support value, and the numbers lower than 50 were not shown. The samples marked with ‘○’ were downloaded from GenBank, and others were newly generated for this analysis. The number of each sample is listed in S1 Table. (TIF) [file pone.0163664.s004.tif]

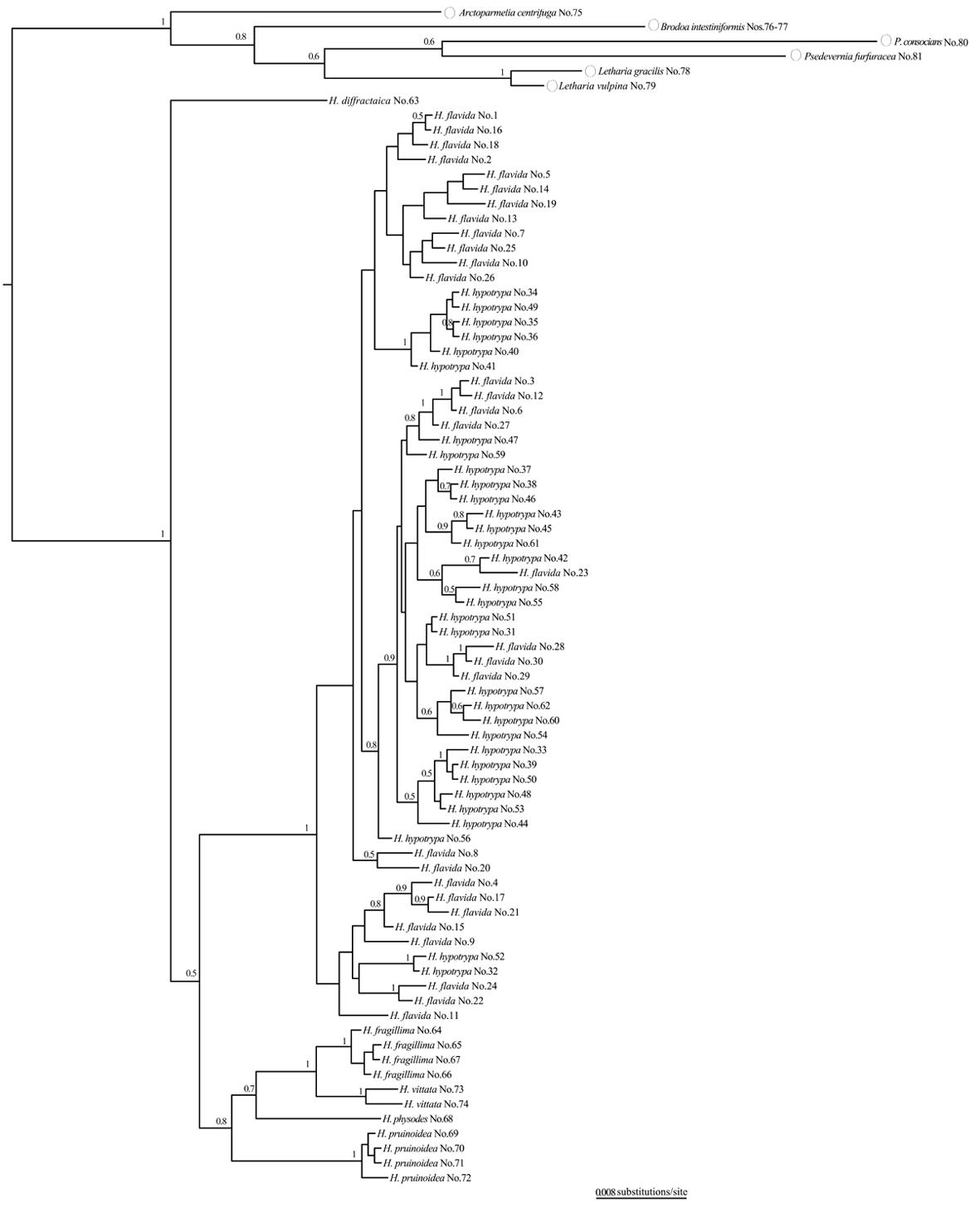

Supplement: S5 Fig — The numbers in each node represents posterior probability value, and the numbers lower than 0.5 were not shown. The samples marked with ‘○’ were downloaded from GenBank, and others were newly generated for this analysis. The number of each sample is listed in S1 Table. (TIF) [file pone.0163664.s005.tif]

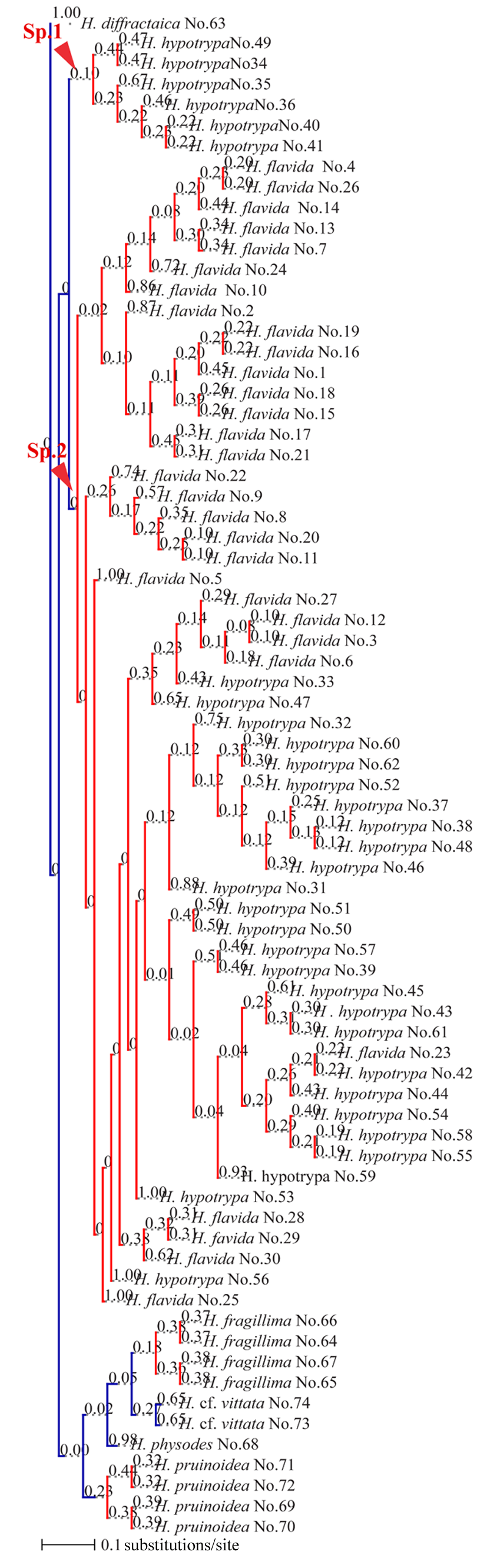

Supplement: S6 Fig — The numbers in each node represents support value. The red color indicates the PTP suggested species, while blue for uncertain. Two main groups suggested here were corresponding to two species (Sp.1 & Sp.2). (TIF) [file pone.0163664.s006.tif]

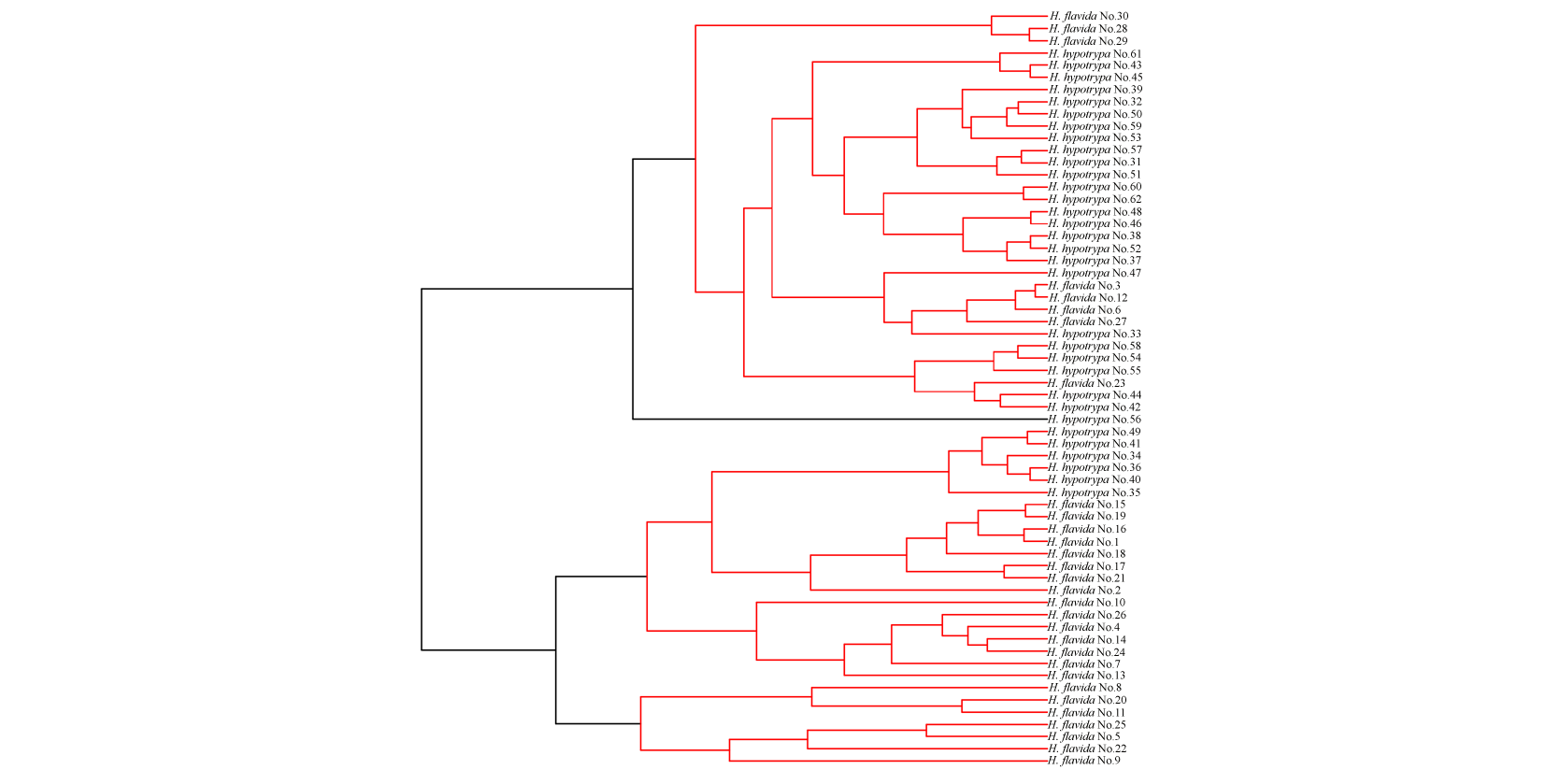

Supplement: S7 Fig — The separated species or populations were indicated by the black lines, while the red line showed the individuals within each species or populations. (TIF) [file pone.0163664.s007.tif]
