# Supplementary material for: Limitations of Species Delimitation Based on Phylogenetic Analyses: A Case Study in the Hypogymnia hypotrypa Group (Parmeliaceae, Ascomycota)
Source: PLoS One. 2016 Nov 9;11(11):e0163664. doi: 10.1371/journal.pone.0163664 (PMC5102465; doi:10.1371/journal.pone.0163664)
Supplement: S1 Table — (DOC) [file pone.0163664.s008.doc]

**S1 Table. Specimens used for DNA extraction and sequences used in this study. Genetic groups corresponds to the results of the Bayesian clustering analysis implemented in the program STRUCTURE: – ‘cluster 1’ generally corresponded to specimens identified as *H. flavida* and the majority of specimens in ‘cluster 2’ were identified as *H. hypotrypa*. All individuals with membership probabilities <0.80 are labeled as ‘admixed’ here.**

| **No.** | **Species** | **Locality, voucher specimens** | **Altitude (m)** | **GenBank no. of ITS sequences** | **GenBank no. of GPD sequences** | **GenBank no. of MCM7 sequences** | **Genetic group** |
| --- | --- | --- | --- | --- | --- | --- | --- |
| 1 | *H*. *flavida* | Shaanxi,N33°34'12" E107°28'12", X.L.Wei 1656 | 3380 | EU293915 | EU306522 | - | ‘cluster 1’ |
| 2 | *H*. *flavida* | Shaanxi,N33°34'12" E107°28'12", X.L.Wei 4064 | 3450 | EU293916 | EU306523 | - | ‘cluster 1’ |
| 3 | *H*. *flavida* | Shaanxi,N33°34'48" E107°27'36", X.L.Wei W11188 | 3488 | JQ836571 | - | - | ‘cluster 2’ |
| 4 | *H*. *flavida* | Shaanxi,N33°29'24" E108°21'36", X.L.Wei W11042 | 2400 | JQ836569 | JQ846455 | KP076651 | ‘cluster 1’ |
| 5 | *H*. *flavida* | Shaanxi,N33°30'36" E108°27'36", X.L.Wei W11014 | 2833 | JQ836570 | JQ846456 | KJ690174 | ‘cluster 1’ |
| 6 | *H*. *flavida* | Shaanxi,N33°34'12"E107°27', X.L.Wei W11236 | 3620 | JQ836572 | JQ846452 | KJ690182 | ‘cluster 2’ |
| 7 | *H*. *flavida* | Yunnan,N26°22'12"E99°25'48", T.Zhang YN2011017 | 3859 | JQ836578 | JQ846454 | KP076652 | ‘cluster 1’ |
| 8 | *H*. *flavida* | Yunnan,N25°20'24"E100°7'48", X.L.Wei 6080 | 3860 | EU293917 | - | - | ‘cluster 1’ |
| 9 | *H*. *flavida* | Yunnan,N25°20'24"E100°7'48", X.L.Wei 6084 | 3860 | EU293918 | - | - | ‘cluster 1’ |
| 10 | *H*. *flavida* | Yunnan,N25°20'24"E100°7'48", X.L.Wei 6095 | 3560 | EU293919 | EU306524 | - | ‘admixed’ |
| 11 | *H*. *flavida* | Yunnan,N25°20'24"E100°7'48", X.L.Wei 6101 | 3860 | EU293920 | EU306525 | - | ‘cluster 1’ |
| 12 | *H*. *flavida* | Shaanxi,N33°34'12"E107°27'36", X.L.Wei W11231 | 3620 | JQ886490 | JQ886491 | KJ690181 | ‘cluster 2’ |
| 13 | *H. flavida* | Shaanxi,N33°29'24"E108°21'36", X.L.Wei W11037 | 2400 | KJ690216 | - | - | ‘cluster 1’ |
| 14 | *H. flavida* | Shaanxi,N33°30'36"E108°27'36", X.L.Wei W11003 | 2833 | KJ690217 | KJ690194 | KJ690175 | ‘cluster 1’ |
| 15 | *H. flavida* | Shaanxi,N33°34'48"E107°27'36", X.L.Wei W11187 | 3627 | KJ690219 | KJ690195 | KJ690176 | ‘cluster 1’ |
| 16 | *H. flavida* | Shaanxi,N33°34'12"E107°27', X.L.Wei W11220 | 3624 | KJ690240 | KJ690197 | KJ690178 | ‘cluster 1’ |
| 17 | *H. flavida* | Shaanxi,N33°35'24"E107°28'48", X.L.Wei W11151 | 3485 | KJ690221 | KJ690198 | KJ690179 | ‘cluster 1’ |
| 18 | *H. flavida* | Shaanxi,N33°34'48"E107°27'36", X.L.Wei W11182 | 3473 | KJ690222 | KJ690199 | KJ690180 | ‘cluster 1’ |
| 19 | *H. flavida* | Shaanxi,N33°34'48"E107°27'36", X.L.Wei W11191 | 3473 | KJ690220 | KJ690196 | KJ690177 | ‘cluster 1’ |
| 20 | *H. flavida* | Yunnan,N26°22'12"E99°25'48", T. Zhang YN2011022 | 3859 | KJ690232 | KJ690200 | - | ‘cluster 1’ |
| 21 | *H. flavida* | Shaanxi,N33°35'24"E107°28'48", X.L.Wei W11159 | 3485 | KJ690218 | - | - | ‘cluster 1’ |
| 22 | *H. flavida* | Tibet,N30°1'48''E95°6'36", Wei &Wang XZ2014472 | 2903 | KP178749 | KP178733 | KP178763 | ‘cluster 1’ |
| 23 | *H. flavida* | Tibet,N30°1'48''E95°6'36", Wei & Wang XZ2014467 | 2903 | KP178750 | KP178734 | KP178764 | ‘admixed’ |
| 24 | *H. flavida* | Tibet,N30°1'48''E95°6'36", Wei & Wang XZ2014452 | 2903 | KP178751 | KP178735 | KP178765 | ‘cluster 1’ |
| 25 | *H. flavida* | Yunnan,N28°20'6.1"E99°5'38.5", Li & Jiang YN201408577 | 4177 | KP178752 | KP178736 | KP178766 | ‘admixed’ |
| 26 | *H. flavida* | Yunnan,N28°20'6.1"E 99°5'38.5", Li & Jiang YN201408573 | 4177 | KP178753 | KP178737 | KP178767 | ‘cluster 1’ |
| 27 | *H. flavida* | Sichuan,N29°49'23.1"E102°3'11.3", Jiang & Li SC201408993 | 2916 | KP178748 | KP178738 | KP178768 | ‘cluster 2’ |
| 28 | *H. flavida* | Taiwan,N24°4'48''E121°10'12'', L.S. Wang & X.Y. Wang 52252 | 3303 | KU187958 | KU187953 | KU187963 | ‘cluster 2’ |
| 29 | *H. flavida* | Taiwan,N24°4'48''E121°10'12'', L.S. Wang & X.Y. Wang 52235 | 3303 | KU187957 | KU187952 | KU187962 | ‘cluster 2’ |
| 30 | *H. flavida* | Taiwan,N24°4'48''E121°10'12'', L.S. Wang & X.Y. Wang 52203 | 3174 | KU187956 | KU187951 | KU187961 | ‘cluster 2’ |
| 31 | *H. hypotrypa* | Russia,N46°38'20"E142°56'44", S. Tchabanenko3 | 65 | KU187959 | KU187954 | KU187964 | ‘cluster 2’ |
| 32 | *H. hypotrypa* | Russia,N47°0'27" E142°47'18", S. Tchabanenko4 | 446 | KU187960 | KU187955 | KU187965 | ‘admixed’ |
| 33 | *H. hypotrypa* | Shaanxi,N33°35'24" E107°28'48", X.L.Wei W11160 | 3485 | KJ690223 | KJ690201 | KJ690184 | ‘cluster 2’ |
| 34 | *H*. *hypotrypa* | Shaanxi,N33°34'12" E107°28'12", X.L.Wei 1693 | 2950 | EU293925 | EU306528 | - | ‘cluster 1’ |
| 35 | *H*. *hypotrypa* | Shaanxi,N33°34'12" E107°28'12", X.L.Wei 1695 | 2950 | EU293926 | EU306529 | - | ‘cluster 1’ |
| 36 | *H*. *hypotrypa* | Shaanxi,N33°34'12" E107°28'12", X.L.Wei 4003 | 3013 | EU293927 | EU306530 | - | ‘cluster 1’ |
| 37 | *H*. *hypotrypa* | Shaanxi,N33°34'12" E107°28'12", X.L.Wei 4004 | 3020 | EU293928 | EU306531 | - | ‘cluster 2’ |
| 38 | *H. hypotrypa* | Shaanxi,N33°34'12" E107°28'12", X.L.Wei 4066 | 3420 | EU293929 | EU306532 | - | ‘cluster 2’ |
| 39 | *H. hypotrypa* | Shaanxi,N34°E107°28'48", X.L.Wei W11124 | 2936 | JQ836574 | JQ846457 | KJ690188 | ‘cluster 2’ |
| 40 | *H. hypotrypa* | Shaanxi,N34°E107°28'48", X.L.Wei W11129 | 2940 | Q836575 | - | - | ‘cluster 1’ |
| 41 | *H. hypotrypa* | Shaanxi,N34°E107°28'48", X.L.Wei W11135 | 2755 | JQ836573 | JQ846453 | KJ690183 | ‘cluster 1’ |
| 42 | *H. hypotrypa* | Tibet,N29°33'E93°9', X.L.Wei 1079 | 3500 | EU293922 | - | - | ‘cluster 2’ |
| 43 | *H. hypotrypa* | Tibet,N29°33'E93°9', X.L.Wei 1115 | 3500 | EU293923 | EU306526 | - | ‘cluster 2’ |
| 44 | *H. hypotrypa* | Tibet,N29°31'48"E95°27', X.L.Wei 1637 | 3372 | EU293924 | EU306527 | - | ‘cluster 2’ |
| 45 | *H. hypotrypa* | Yunnan,N25°20'24" E102°15'36", X.L.Wei 6042 | 3200 | EU293930 | EU306533 | - | ‘cluster 2’ |
| 46 | *H. hypotrypa* | Shaanxi,N33°34'48" E107°27'36", X.L.Wei W11228 | 3508 | KJ690227 | KJ690205 | KJ690189 | ‘cluster 2’ |
| 47 | *H. hypotrypa* | Shaanxi,N34°E107°28'48", X.L.Wei W11101 | 2800 | KJ690228 | KJ690206 | KJ690190 | ‘cluster 2’ |
| 48 | *H. hypotrypa* | Shaanxi,N33°35'24" E107°28'48", X.L.Wei W11163 | 3480 | KJ690229 | KJ690207 | KJ690191 | ‘cluster 2’ |
| 49 | *H. hypotrypa* | Shaanxi,N34°E107°28'48", X.L.Wei W11076 | 2697 | KJ127505 | KJ127502 | KJ690186 | ‘cluster 1’ |
| 50 | *H. hypotrypa* | Shaanxi,N34°E107°28'48", X.L.Wei W11117 | 2908 | KJ127504 | KJ127501 | KJ690185 | ‘cluster 2’ |
| 51 | *H. hypotrypa* | Japan,N35°52'58",E138°10'25", 09091210 | 1800 | KP076648 | KP076646 | KP076654 | ‘cluster 2’ |
| 52 | *H. hypotrypa* | Japan,N35°51'40",E138°11'12", 12100615 | 2000 | KP076649 | KP076647 | KP076655 | ‘admixed’ |
| 53 | *H. hypotrypa* | Shaanxi,N34°E107°28'48", X.L.Wei W11137 | 2955 | KJ127506 | KJ127503 | KJ690187 | ‘cluster 2’ |
| 54 | *H. hypotrypa* | Tibet,N29°27' E94°26'24'', Wei & Wang XZ2014237 | 3258 | KP178754 | KP178740 | KP178769 | ‘cluster 2’ |
| 55 | *H. hypotrypa* | Tibet, N29°27' E94°26'24'', Wei & Wang XZ2014235 | 3258 | KP178755 | KP178741 | KP178770 | ‘cluster 2’ |
| 56 | *H. hypotrypa* | Tibet, N29°27' E94°26'24'', Wei & Wang XZ2014244 | 3258 | KP178756 | KP178742 | KP178771 | ‘admixed’ |
| 57 | *H. hypotrypa* | Sichuan,N30°7'34.6''E100°42'27'', Li & Jiang SC201408834 | 4190 | KP178757 | KP178743 | KP178772 | ‘cluster 2’ |
| 58 | *H. hypotrypa* | Sichuan,N30°7'34.6''E100°42'27'', Li & Jiang SC201408835 | 4190 | KP178758 | KP178744 | KP178773 | ‘cluster 2’ |
| 59 | *H. hypotrypa* | Sichuan,N30°7'34.6''E100°42'27'', Li & Jiang SC201408833 | 4190 | KP178759 | KP178745 | KP178774 | ‘cluster 2’ |
| 60 | *H. hypotrypa* | Yunnan,N26°59'26.7''E100°10'32.1'', Jiang & Li YN201408266 | 3281 | KP178760 | KP178739 | KP178775 | ‘cluster 2’ |
| 61 | *H. hypotrypa* | Yunnan,N26°59'26.7''E100°10'32.1'', Jiang & Li YN201408271 | 3281 | KP178761 | KP178746 | KP178776 | ‘cluster 2’ |
| 62 | *H. hypotrypa* | Yunnan,N26°59'26.7''E100°10'32.1'', Jiang & Li YN201408258 | 3281 | KP178762 | KP178747 | KP178777 | ‘cluster 2’ |
| 63 | *H. diffractaica* | Yunnan,N26°37'E99°43, T. Zhang YN2011009 |  | KJ690233 | KJ690214 | KP076650 | - |
| 64 | *H. fragillima* | Shaanxi,N34°E107°28'48'', X.L.Wei W11119 |  | KJ690238 | - | - | - |
| 65 | *H. fragillima* | Shaanxi,N34°E107°28'48'', X.L.Wei W11141 |  | KJ690231 | KJ690209 | KP076653 | - |
| 66 | *H. fragillima* | Shaanxi,N33°30'36" E108°27'36", X.L.Wei W11002 |  | KJ690239 | KJ690193 | - | - |
| 67 | *H. fragillima* | Shaanxi,N33°35'24" E107°28'48", X.L.Wei W11152 |  | KJ690230 | KJ690215 | - | - |
| 68 | *H*.*physodes* | Shaanxi,N34°E107°28'48'', X.L.Wei W11200 |  | JQ836576 | JQ846458 | - | - |
| 69 | *H. pruinoidea* | Shaanxi,N34°E107°28'48'', X.L.Wei W11092 |  | KJ690236 | KJ690211 | - | - |
| 70 | *H. pruinoidea* | Shaanxi,N34°E107°28'48'', X.L.Wei W11148 |  | KJ690237 | KJ690212 | - | - |
| 71 | *H. pruinoidea* | Shaanxi,N34°E107°28'48'', X.L.Wei W11139 |  | KJ690234 | KJ690208 | KJ690192 | - |
| 72 | *H. pruinoidea* | Shaanxi,N34°E107°28'48'', X.L.Wei W11143 |  | KJ690235 | KJ690210 | - | - |
| 73 | *H. vittata* | Shaanxi,N34°E107°28'48'', X.L.Wei W11140 |  | JQ836577 | JQ846459 | KP076656 | - |
| 74 | *H. vittata* | Shaanxi,N33°34'12" E107°28'12", X.L.Wei 1940 |  | EU293931 | EU306535 | - | - |
| 75* | *Arctoparmelia centrifuga* |  |  | AY581054 |  |  |  |
| 76* | *Brodoa intestiniformis* |  |  | DQ980002 | AY340870 | - |  |
| 77* | *Brodoa intestiniformis* |  |  | - | - | KP938770 |  |
| 78* | *Letharia gracilis* |  |  | KJ565862 | - | - |  |
| 79* | *Letharia vulpina* |  |  | KJ565924 | - | - |  |
| 80* | *Pseudevernia consocians* |  |  | HQ650614 | - | - |  |
| 81* | *Pseudevernia furfuracea* |  |  | GU300791 | - | - |  |

Note: The species name of Nos.73-74 when submitting DNA sequences to GenBank were *Hypogymnia subduplicata*, which actually were the wrong identification of *H.* cf. *vittata*. *The sequences were downloaded from GenBank.
